# PANoptosis: a new insight into the mechanism of ischaemia–reperfusion injury

**DOI:** 10.1093/burnst/tkaf026

**Published:** 2025-04-10

**Authors:** Huapei Song, Guangping Liang, Fengjun Wang

**Affiliations:** Institute of Burn Research, The First Affiliated Hospital of Army Medical University (The Third Military Medical University), State Key Laboratory of Trauma and Chemical Poisoning, Gaotanyan Street, Shapingba Disdrict, Chongqing 400038, China; Institute of Burn Research, The First Affiliated Hospital of Army Medical University (The Third Military Medical University), State Key Laboratory of Trauma and Chemical Poisoning, Gaotanyan Street, Shapingba Disdrict, Chongqing 400038, China; Institute of Burn Research, The First Affiliated Hospital of Army Medical University (The Third Military Medical University), State Key Laboratory of Trauma and Chemical Poisoning, Gaotanyan Street, Shapingba Disdrict, Chongqing 400038, China

**Keywords:** PANoptosis, PANoptosome, Apoptosis, Necroptosis, Pyroptosis, Ischaemia–reperfusion injury

## Abstract

Programmed cell death, which occurs via modes such as apoptosis, necroptosis, and pyroptosis, is an important mechanism for host defence against pathogens and inflammation-mediated immune responses. Recently, interactions between various types of cell death have gradually been discovered. PANoptosis is a newly discovered mode of programmed cell death that involves apoptosis, necroptosis, and pyroptosis and is closely related to many diseases. Ischaemia–reperfusion injury (IRI) is common in patients with blood circulation disorders such as those related to burns, traumatic shock, surgery, organ transplantation, and thrombus. However, the literature on the role of PANoptosis in IRI is limited. Herein, we systematically described the emergence of PANoptosis as a cell death mode, clinical evidence of its occurrence, the molecular mechanisms of PANoptosis, and its role in IRI. This study is expected to provide novel approaches for the prevention and treatment of tissue and organ IRI after severe burns.

## Highlights

- This is the first to review the role of PANoptosis in ischaemia–reperfusion injury (IRI), which might be helpful to clarify the function and mechanism of PANoptosis in tissue IRI after severe burns.
- This review systematically described the emergence, clinical evidence, molecular mechanisms of PANoptosis, and its role in IRI.

## Background

Cell death is an important mechanism for host defense against pathogens and inflammation-mediated immune responses. Under the influence of a variety of external stimuli and the intracellular environment, programmed cell death can be executed through different death methods via specific genetic encoding mechanisms, including apoptosis, pyroptosis, necroptosis, ferroptosis, and autophagy [1]. The molecular mechanisms of the initiation, transduction, and execution of apoptosis, pyroptosis, and necroptosis have been elucidated. Pyroptosis is mediated by inflammasomes and is characterized by the formation of caspase-1 (CASP1)-dependent pores on the plasma membrane, cell lysis, and the release of inflammatory contents. Apoptosis is considered a relatively mild nonlytic mode of cell death and is a CASP-dependent non-inflammatory process characterized by the formation of apoptosomes. Necroptosis is associated with a variety of cytokines and is mediated mainly by receptor-interacting protein kinase 3 (RIPK3), which is characterized by cell swelling and membrane destruction [2]. Most early cell death studies focused on a single death mechanism. With increasing research on cell death, interactions between various cell death signalling pathways have gradually been discovered. In 2016, Kanneganti *et al*. reported that the internal proteins nucleoprotein and polymerase basic protein 1 of influenza A virus (IAV) can bind to Z-DNA binding protein 1 (ZBP1) to promote the activation of the NLR family pyrin domain-containing protein 3 (NLRP3) inflammasome and trigger simultaneous apoptosis, necrosis, apoptosis, and pyroptosis in mouse bone marrow-derived macrophages through the RIPK1–RIPK3–CASP8 pathway, triggering inflammation [3]. Subsequent studies revealed complex cross-talk among various types of cell death, and several modes of cell death can coexist in pathological environments, share overlapping mechanisms, and act as ‘backup’ death strategies to ensure organism homeostasis [4–6]. In 2019, Kanneganti *et al*. named this complex mode of cell death PANoptosis, which is activated by specific triggers and regulated by the PANoptosome; it has the key characteristics of pyroptosis, apoptosis, and necroptosis, but its effects cannot be explained by the occurrence of any of these individual types of programmed cell death alone [7, 8]. PANoptosis has unique regulatory methods and is closely related to many diseases, including infection, sterile inflammation, acute lung injury (ALI), acute respiratory distress syndrome, tissue ischaemia–reperfusion injury (IRI), cancers, and metabolic diseases [9–13]. IRI is common in patients with blood circulation disorders such as those related to burns, traumatic shock, surgery, organ transplantation, and thrombus. The main injury mechanisms are increased aerobic free radicals, increased mitochondrial permeability, and increased expression of cytokines or adhesion molecules [14–16]. In this review, we mainly focused on the emergence, molecular mechanisms, and clinical evidence of PANoptosis and its role in IRI.

## Review

### Emergence of PANoptosis

**Figure 1:**
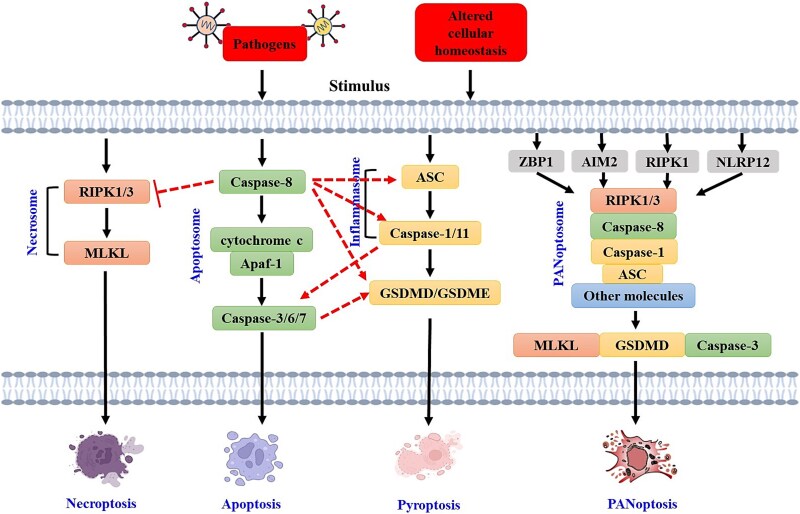
PCD pathways. Pathogens or dysregulation of cellular homeostasis can cause the activation of different PCD pathways. Pyroptosis, apoptosis, necroptosis, and PANoptosis are four distinct PCD pathways. PANoptosomes include sensors, adaptors, and catalytic effectors bound by interacting domains; these structures assemble to elicit PANoptosis. Specific sensors (e.g. ZBP1, AIM2, RIPK1, and NLRP12) are activated by the trigger, which initiates the assembly of the PANoptosome-containing molecules required to activate downstream PCD effectors, including GSDMD, CASP3, and MLKL. *RIPK* receptor-interacting protein kinase, *MLKL* mixed-lineage kinase domain-like proteins, *ASC* apoptosis-associated speck-like protein, *AIM* absent in melanoma, *NLRP* NLR family pyrin domain-containing protein

Conventionally, programmed cell death (PCD) pathways have been categorized into lytic and nonlytic forms, including pyroptosis, necroptosis, and apoptosis. Pyroptosis is an inflammatory PCD pathway with distinct morphological characteristics, such as cell swelling, DNA fragmentation, and plasma membrane rupture. Pyroptotic cell death is mediated through the assembly of a multiprotein signalling complex called an inflammasome [17, 18]. Apoptosis is classified into intrinsic and extrinsic pathways and has morphological features, including cell shrinkage, nuclear condensation, DNA fragmentation, and membrane blebbing [19]. It is regulated by the complex apoptosome, and a typical example is that APAF-1 senses mitochondrial-derived cytochrome C and interacts with CASP9 through the CARD domain [20]. Morphologically, necroptosis is characterized by organelle swelling, loss of plasma membrane integrity, and cell lysis. Under the inhibition of CASP8 activity, RIPK3 and RIPK1 form a necrosome that mediates necroptosis through the RHIM–RHIM interaction [21]. Recent studies highlighted the cross-talk and redundancies among pyroptosis, apoptosis, and necroptosis. Researchers have reported that the loss of a single component that drives pyroptosis, apoptosis, or necroptosis is often insufficient to prevent lytic cell death, indicating redundancies among upstream regulators. Nonetheless, inhibiting key components of all three pathways together results in complete protection against cell death [3]. On the basis of the extensive cross-talk between PCD pathways, an integrated cell death modality called ‘PANoptosis’ is formed, and the effects cannot be accounted for by pyroptosis, apoptosis, or necroptosis alone (Figure 1). PANoptosis is a unique innate immune inflammatory PCD pathway that is mechanistically regulated by multifaceted PANoptosome complexes with morphological characteristics, including condensed chromatin, fragmented DNA, shrinking cells, and compromised membranes. PANoptosis can be modulated by diverse PANoptosome complexes that are assembled from pyroptotic, apoptotic, and necroptotic mediators [11]. The protein complex simultaneously modulates the three modes of cell death. The distinct features of PANoptosis compared with traditional cell death pathways (apoptosis, necroptosis, and pyroptosis) are shown in Table 1. Multiple PANoptosome sensors and regulators are required for eliciting PCD. For example, the PANoptosome was shown to contain RIPK1, RIPK3, apoptosis-associated speck-like protein (ASC), NLRP3, ZBP1, CASP1, CASP6, and CASP8, which are components of the PANoptosome in response to IAV infection [22, 23].

**Table 1:** The features of pyroptosis, apoptosis, necroptosis, and PANoptosis

	Pyrotosis	Apoptosis	Necroptosis	PANoptosis
Morphology	Cell swelling DNA Fragmentation Membrane rupture	Cell shrinkage Nuclear condensation DNA fragmentation Membrane blebbing	Organelle swelling Loss of plasma membrane integrity Cell lysis	Condensed chromatin DNA fragmentation Cell shrinkage Compromised membranes
Immunology	Pro-inflammatory	Anti-inflammatory	Pro-inflammatory	Pro-inflammatory
Actuator	Inflammasome	Apoptosome	Necrosome	PANoptosome
Key proteins	Caspase-1 IL-1β NLRP3 ASC GSDMD	Bcl-2 BAX Caspase-3	RIPK1/3 MLKL Caspase-8	RIPK1/3 NLRP3 ASC Caspase-1/3/8 MLKL GSDMD

*RIPK* receptor-interacting protein kinase, *MLKL* mixed-lineage kinase domain-like proteins, *ASC* apoptosis-associated speck-like protein, *AIM* absent in melanoma, *NLRP* NLR family pyrin domain-containing protein

### Molecular mechanisms of PANoptosis

#### PANoptosome

PANoptosis is a newly discovered cell death pathway. The assembly and formation of the PANoptosome is key to achieving multipathway cross-talk [24, 25]. PANoptosomes and inflammasomes have similar structures, with the core protein structure composed of three parts: sensor proteins, adapter proteins, and effector proteins (Figure 1). The sensor proteins that participate in the formation of the PANoptosome include ZBP1, absent in melanoma 2 (AIM2), RIPK1, and RIPK12. The adapter proteins include ASC and Fas-associated protein with death domain (FADD). The effector proteins include the proteolytic enzymes CASP8, CASP1, CASP3, and CASP6; the RIP homotypic interaction motif (RHIM) domain-containing proteins RIPK1 and RIPK3; the pore-forming proteins gasdermin D (GSDMD) and gasdermin E; and mixed-lineage kinase domain-like proteins (MLKL) [26, 27]. The PANoptosis signal transduction process usually starts with the detection of endogenous or exogenous signals by specific sensor proteins. The downstream adapter proteins receive signals recognized by sensor proteins and transduce signals to effector proteins such as CASP, RIPK, and MLKL through protein–protein interactions. CASP1 cleaves GSDMD to form pores on the cell membrane and cleaves pro-IL-1β and pro-IL-18 to form mature IL-1β and IL-18; CASP8 activates CASP3 and CASP7; RIPK1 interacts with RIPK3 to activate RIPK3; and RIPK3 subsequently phosphorylates MLKL, eventually simultaneously inducing programmed cell death modes such as pyroptosis, apoptosis, and necroptosis and promoting the release of inflammatory cytokines [28]. The PANoptosome also contains master regulators of three modes of cell death that can induce cell death and sense pathogen-associated molecular patterns (PAMPs), damage-associated molecular patterns (DAMPs), or other risk factors [24, 29]. However, no consensus has been reached regarding the criteria for classification of the death mode according to these components. Under different stimuli, proteins play different roles in cells, which is one of the reasons why PANoptosis is a complex process that is difficult to study. For example, necroptosis requires RIPK1, which has kinase activity, to act as a catalytic effector molecule, whereas in transforming growth factor-β-activated kinase 1 (TAK1)-deficient cells, the scaffolding of kinase-inactive RIPK1 is required for NLRP3 inflammasome activation and cell death, suggesting that RIPK1 may also act as an adapter protein [22]. In summary, a sensor recognizes different PAMPs or DAMPs in an associative manner to initiate PANoptosome assembly through the homotype or heterotype interaction of adapter domains and acts as a molecular scaffold to activate the key molecules of three cell death pathways, i.e. apoptosis, pyroptosis, and necroptosis, and inflammatory cells through different catalytic effectors.

#### Key molecules in PANoptosis

##### AIM2

AIM2 is a classic sensor protein located in the cytoplasm. AIM2 cooperates with CASP1 and ASC to form the AIM2 inflammasome, which recognizes infection by pathogenic microorganisms and binds to cytoplasmic double-stranded DNA to initiate the innate immune response and induce inflammation [30]. Lee *et al*. reported that in bone marrow-derived macrophages (BMDM) cells and animal models infected with herpes simplex virus and Francisella, AIM2, pyrin, ZBP1, ASC, CASP1, CASP8, RIPK3, RIPK1, ASC, and FADD together form a PANoptosome that can drive the release of inflammatory factors [9].

##### ZBP1

ZBP1, also known as DNA-dependent activator of interferon-regulatory factor (DAI) or DLM-1, is a key mediator of NLRP3 inflammasome activation and PANoptosis under certain conditions [11]. ZBP1 is an innate immune receptor that contains a RHIM that mediates cell death and Z-α1 and Z-α2 domains that bind to Z-nucleic acid, which can sense foreign pathogens such as viral nucleic acids and fungi, direct the synthesis of cell death signals, and induce cell apoptosis and the release of inflammatory factors. Loss of the ZBP1 and Z-α2 domains results in reduced NLRP3 activation (pyroptosis); reduced cleavage of CASP3, CASP8, and CASP7 (apoptosis); and reduced MLKL phosphorylation (necroptosis) [31, 32]. In addition, ZBP1 also activates RIPK1 to drive NF-κB activation and inflammation in response to IAV infection. Knocking out ZBP1 completely inhibited IAV-induced NLRP3 inflammasome activation and cytokine release [33].

##### The CASP family

The caspase family plays key roles in the control of host cell death, the innate immune response and homeostasis [34]. The caspase family can be divided into inflammatory CASPs (CASP1, CASP4, CASP5, and CASP11) and apoptotic CASPs (CASP3, CASP6, and CASP10) [35]. During IAV infection, CASP6 binds to RIPK3 to enhance the interaction between RIPK3 and ZBP1, thereby promoting ZBP1–PANoptosome assembly and driving PANoptosis body activation and cell death [23]. In addition to playing an enzymatic role in cell apoptosis and necroptosis, CASP8 can act as a scaffolding protein and, together with MLKL, regulate the activation of the NLRP3 inflammasome in macrophages to induce pyroptosis. CASP8 can also form a protein complex consisting of RIPK1 and RIPK3 and interact with other related proteins through the RHIM and DD to mediate cell pyroptosis and necroptosis [36, 37]. In addition, CASP1, CASP3, and CASP7 are involved in the regulation of PANoptosis [27].

##### TAK1

TAK1 is a core regulator of innate immunity, cell death, inflammation, and cellular homeostasis and is involved in pro-cell survival signal transduction [38]. TAK1 inactivation or deletion leads to the loss of cell homeostasis, the release of RIPK1 kinase activity-dependent inflammatory signals, and the activation of the NLRP3 inflammasome and PANoptosis [22]. Under physiological conditions, TAK1 can inhibit the phosphorylation of RIPK1 and block the activation of PANoptosis [39]. BMDMs lacking TAK1 undergo spontaneous cell death, and TAK1-deficient cells form a PANoptosome consisting of RIPK1 and CASP8 through receptor interactions, thus triggering RIPK1-dependent PANoptosis [7]. In Yersinia-infected cells, the inhibition or knockout of TAK1 promotes the formation of RIPK1-PANoptosomes containing RIPK1, ASC, and CASP8 and induces spontaneous inflammatory cell death. This PANoptosome promotes FADD–CASP8-dependent apoptosis through the RIPK3-mediated phosphorylation of MLKL, leading to necroptosis, as well as NLRP3 inflammasome activation and pyroptosis [40]. In addition, TAK1-deficient mice exhibit an acute myeloid leukaemia-like phenotype and are highly sensitive to inflammatory septic shock; the inactivation of RIPK1 kinase can partially protect these mice through the inhibition of PANoptosis [41].

### Clinical evidence of PANoptosis

Recent studies have demonstrated the participation of PANoptosis in several diseases. Studies that have analysed the molecular changes in PANoptosis-related genes in cancer have reported distinct cell clusters that exhibit unique immune microenvironments, biological functions, viability rates, and chemotherapy drug sensitivity. These findings highlight the therapeutic and prognostic value of PANoptosis, revealing a new area for exploration. Pan *et al*. established three different patterns of PANoptosis by analysing samples from 1316 gastric cancer patients, each with unique clinical, molecular, and immunological features that can be quantified by a scoring system called the PANscore. The low PANscore group had a greater response rate to immunotherapy and a better prognosis than the high PANscore group. Thus, the PANscore can be used to guide treatment decision making for gastric cancer [42]. Ouyang *et al*. conducted a comprehensive bioinformatics analysis to evaluate both the expression and mutation patterns of PANoptosis-related genes in hepatocellular carcinoma (HCC) patients, which revealed the promise of PANoptosis-based molecular clustering and prognostic signatures in predicting patient survival and discerning the intricacies of the tumour microenvironment within the context of HCC [43]. Yi *et al*. compared PANoptosis pathway gene expression levels between tumour and normal adjacent tissues from patients with prostate cancer (PRAD) and evaluated the genomic, transcriptomic, and clinical features of the PANoptosis signature in PRAD. By constructing a PANoptosis signature, the prognosis and immunotherapeutic response of patients can be predicted [44]. The PANoptosis pathway has also been studied in diseases other than cancer. For example, Wang *et al*. constructed a PANoptosis gene set and revealed significant activation of PANoptosis in ulcerative colitis patients on the basis of multiple transcriptome profiles of intestinal mucosal biopsies from the Gene Expression Omnibus database. They hypothesized that IRF1, as a TF, promotes PANoptosome multicomponent expression, activates PANoptosis, and then induces excessive intestinal epithelial cell death [45]. Yang *et al*. conducted a bioinformatics analysis of online single-cell RNA sequence (scRNA-seq) and bulk RNA-seq datasets to explore the potential of PANoptosis as an indicator of COVID-19 severity. The degree of PANoptosis in bronchoalveolar lavage fluid and peripheral blood mononuclear cells indicates the severity of COVID-19 [46]. On the basis of findings of PANoptosis in abdominal aortic aneurysm (AAA) clinical samples, Li *et al*. reported that the inhibition of tumour necrosis factor-α and interleukin-1β can reduce PANoptosis in vascular smooth muscle cells and thus alleviate the process of AAA [47]. Shi *et al*. demonstrated that the occurrence and progression of COPD are closely related to PANoptosis. They also identified the PANoptosis-related gene PYCARD associated with COPD inflammation, immune responses, the C-type lectin receptor signalling pathway, and the metabolic pathway [48]. Lan *et al*. employed bioinformatics analysis to identify seven key differentially expressed PANoptosis-related genes involved in the pathogenesis of osteoarthritis, which underscored the potential involvement of PANoptosis in immune regulation [49].

### Role of PANoptosis in IRI

**Figure 2:**
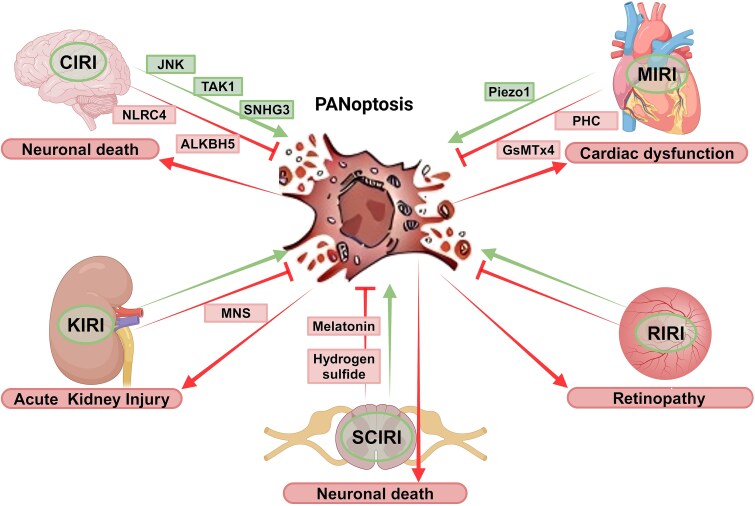
Schematic representations of PANoptosis contributing to tissue damage in different types of ischaemia–reperfusion injuries. *CIRI* cerebral ischaemia–reperfusion injury, *MIRI* myocardial ischaemia–reperfusion injury, *SCIRI* spinal cord ischaemia–reperfusion injury

IRI contributes to morbidity and mortality in a wide range of pathologies, including myocardial infarction, ischaemic stroke, acute kidney injury (AKI), trauma, organ transplantation, and general surgery. An imbalance in metabolic supply and demand within ischaemic organs results in tissue hypoxia and microvascular dysfunction. Subsequent reperfusion further enhances the activation of innate and adaptive immune responses and cell death programmes [50]. The underlying molecular mechanisms of IRI include Wnt, Notch, phosphatidylinositol 3-kinase/protein kinase B, transforming growth factor-β, nuclear factor kappa, bone morphogenetic protein, *N*-methyl-d-aspartic acid receptor-Ca^2+^-Activin A, Hippo-Yes-associated protein, toll-like receptor 4/toll-interleukin-1 receptor domain-containing adapter-inducing interferon-β, and hepatocyte growth factor/mesenchymal–epithelial transition factor [14]. Recent advances in understanding the molecular and immunological consequences of PANoptosis in IRI of different organs may lead to innovative therapeutic strategies for treating patients with ischaemia and reperfusion-associated tissue inflammation and organ dysfunction (Figure 2).

Cerebral ischaemia–reperfusion injury (CIRI) often involves inflammation and immune system activation and can lead to severe brain injury [51, 52]. Zhang *et al*. reported the occurrence of PANoptosis in CIRI [53]. As a key molecule of necroptosis, RIPK3 can interact with the JunN-terminal kinase-mediated inflammatory signalling pathway. This pathway is closely related to ischaemia-induced neuronal apoptosis and pyroptosis [54, 55]. Poh *et al*. reported that during CIRI, the NLRC4 inflammasome interferes with the two components of PANoptosis and blocks thromboxane A synthase/thromboxane A2/thromboxane prostaglandin signalling while simultaneously inhibiting apoptosis and pyroptosis [56]. Wu *et al*. reported that the inhibition of TAK1 attenuates CIRI-induced neuronal death. These findings indicate that TAK1 may serve as an important target in PCD induced by IRI. TAK1 affects the function of microglia and interacts with inflammatory pathways, thereby affecting neuronal apoptosis and pyroptosis [57]. TAK1 also plays an important role in the interaction between neuronal programmed necrosis and apoptosis mediated by RIP3 during CIRI [58]. Through *in vivo* and *in vitro* experiments in mice, Qiu *et al*. determined that cerebral ischaemia–reperfusion suppresses the expression of the m6A demethylase ALKBH5 but increases the expression levels of SNHG3 and PANoptosis-related proteins and that ALKBH5 overexpression and SNHG3 deficiency suppress PANoptosis, indicating that ALKBH5 may exert its protective effect through the inhibition of SNHG3-mediated PANoptosis to reduce IRI [59].

Spinal cord ischaemia–reperfusion injury (SCIRI) is secondary damage caused by primary spinal cord injury and refers to the phenomenon in which, after blood perfusion of the ischaemic spinal cord tissue is restored, structural and functional damage, and even irreversible delayed death of spinal cord neurons, occurs [60, 61]. Xie *et al*. reported that PANoptosis may be the cause of massive neuronal death and paraparesis in SCIRI-induced rats and that hydrogen sulphide and melatonin can provide neuroprotection for the spinal cord through the inhibition of PANoptosis [62, 63].

Myocardial ischaemia–reperfusion injury (MIRI) can lead to further damage to the ultramicroscopic structure, function, metabolism, and electrophysiology of the ischaemic myocardium [64–66]. Cui *et al*. confirmed that in a rat model of MIRI, preconditioning with penehyclidine hydrochloride (PHC) significantly improved cardiac function. The mechanism involves PHC significantly reducing the expression levels of PANoptosis regulatory proteins, possibly through the inhibition of ZBP1 expression [67]. Li *et al*. reported that PANoptosome-related proteins (CASP8, CASP3, CASP1, NLRP3, GSDMD, RIPK1, RIPK3, and MLKL) were highly expressed in mouse hearts during IRI, indicating PANoptosis. GsMTx4, an inhibitor of the mechanosensitive ion channel Piezo1, significantly reduces the development of PANoptosis and attenuates the I/R-mediated decrease in cardiac systolic function and increase in myocardial infarct size. Piezo1 may promote cardiac IRI through the CASP8-mediated PANoptosis of myocardial cells and is expected to become a novel target for the treatment of ischaemic heart disease [68].

Retinal ischaemia–reperfusion injury is an important pathophysiological basis of various ischaemic retinal diseases, such as diabetic retinopathy, glaucoma, and central retinal arteriovenous occlusion, for which existing intervention measures have little effect [69]. Through *in vivo* and *in vitro* retinal neuronal IRI models, Yan *et al*. reported that IRI significantly upregulates the expression of important PANoptosome proteins, such as CASP1, CASP8, and NLRP3, preliminarily confirming the occurrence of PANoptosis in IRI-induced retinal neurons [70].

Kidney ischaemia–reperfusion injury (KIRI) is a common pathophysiological basis of kidney transplantation, partial nephrectomy, and complex cardiovascular surgery, as well as an important cause of AKI and delayed graft function [71, 72]. Uysal *et al*. reported that the administration of 3,4-methylenedioxy-β-nitrostyrene before ischaemia–reperfusion in rat kidneys reduces PANoptosis in cells; NLRP3, the key protein of the PANoptosome, can be selectively inhibited to suppress PANoptosis and thus protect the kidney from KIRI [73].

## Conclusions

As a new mode of programmed cell death, PANoptosis not only involves complex regulatory mechanisms but is also closely related to various diseases. In this review, we systematically described the emergence of PANoptosis, as well as clinical evidence of its occurrence, the molecular mechanisms of PANoptosis and its role in IRI. Although great progress has been made in the study of PANoptosis, many issues remain to be addressed. For example, different pathogens or stimuli can induce PANoptosis via the same receptors, but the detailed mechanisms remain unclear. In addition to the reported components of PANoptosomes, there are likely unidentified molecules that mediate the assembly of PANoptosomes. In the context of PANoptosis, the cross-talk among the key proteins involved in pyroptosis, apoptosis, or necroptosis should be further investigated. PANoptosis has shown beneficial or detrimental effects in different diseases, which is worthy of further research in specific models. NLRP3 inflammasome activation has been reported to play a beneficial role in burn wound healing, burn sepsis, burn-induced ALI, and hypertrophic scarring. To our knowledge, the function and mechanisms of PANoptosis in IRI after severe burns are still unclear. The role of PANoptosis in organ (heart, lung, kidney, etc.) injury after severe burns remains unknown. Whether the assembly of PANoptosomes and the associated changes in protein expression during different stages (resuscitation, infection control, and rehabilitation) of burns should be investigated. The signalling pathway involved in PANoptosis after burns, which might be involved in the targeted treatment of medication, needs to be illustrated. With increasing research on PANoptosis, the structure and regulatory mechanism of PANoptosomes will become clearer; studies on this topic are expected to provide novel approaches for the prevention and treatment of tissue IRI after severe burns.
